# PPARγ activation suppresses chondrocyte ferroptosis through mitophagy in osteoarthritis

**DOI:** 10.1186/s13018-023-04092-x

**Published:** 2023-08-24

**Authors:** Xiang Xue, Tianming Dai, Junyan Chen, Yangyang Xu, Zhenyu Yang, Jian Huang, Wuyan Xu, Siming Li, Qingqi Meng

**Affiliations:** 1 Department of Orthopedics, Guangzhou Red Cross Hospital of Jinan University, Guangzhou, China; 2 Guangzhou Institute of Traumatic Surgery, Guangzhou Red Cross Hospital of Jinan University, Guangzhou, China; 3 Guizhou Medical University, Guiyang, China; 4 Department of Traumatic Orthopedics, The Central Hospital of Xiaogan, Xiaogan, China

**Keywords:** Osteoarthritis, Chondrocyte, PPARγ, Ferroptosis, Pink1, Mitophagy

## Abstract

**Background:**

Osteoarthritis (OA) is a prevalent disease plaguing the elderly. Recently, chondrocyte ferroptosis has been demonstrated to promote the progression of OA. Peroxisome proliferator-activated receptor-γ (PPARγ) is an important factor in maintaining cartilage health. However, the relationship between PPARγ and chondrocyte ferroptosis in OA and its mechanism is completely unclear.

**Methods:**

We established a surgically induced knee OA rat model to investigate PPARγ and chondrocyte ferroptosis in OA. Rat knee specimens were collected for Safranin O/Fast Green staining and immunohistochemical staining after administered orally placebo or pioglitazone (PPARγ agonist) for 4 weeks. We used RSL3 to establish a chondrocyte ferroptosis model cultured in vitro to study the role of PPARγ activation toward ferroptosis, mitochondrial function, and PTEN-induced putative kinase 1 (Pink1)/Parkin-dependent mitophagy. GW9662 (PPARγ antagonist), Mdivi-1 (mitophagy inhibitor), and chloroquine (mitophagy inhibitor) were employed to investigate the mechanism of PPARγ-Pink1/Parkin-dependent mitophagy in the inhibition of ferroptosis.

**Results:**

We found that PPARγ activation by pioglitazone attenuated not only OA but also inhibited the expression of the ferroptosis marker acyl-CoA synthetase long-chain family member 4 (ACSL4) at the same time in rats. Furthermore, in vivo and in vitro data indicated that PPARγ activation restored Pink1/Parkin-dependent mitophagy, improved mitochondrial function, inhibited chondrocyte ferroptosis, and delayed the progression of OA.

**Conclusions:**

The present study demonstrated that PPARγ activation attenuates OA by inhibiting chondrocyte ferroptosis, and this chondroprotective effect was achieved by promoting the Pink1/Parkin-dependent mitophagy pathway.

**Supplementary Information:**

The online version contains supplementary material available at 10.1186/s13018-023-04092-x.

## Background

Osteoarthritis (OA) is the most prevalent degenerative joint disease in the world, and the symptoms are mainly joint pain, stiffness, and limited mobility. Current treatments can only control symptoms. There is no satisfactory way to prevent or reverse the progression of OA, and ultimately it is difficult to avoid arthroplasty in the end [1], which carries a huge financial burden on both individuals and society. Although obesity and mechanical, metabolic, and genetic factors are associated with OA, the exact pathogenesis remains unclear [2].

Ferroptosis is an iron- and oxidative stress-dependent novel form of cell death and is relevant to many diseases [3, 4]. Recently, studies have demonstrated the contribution of chondrocyte ferroptosis to the progression of OA [5, 6]. Research evidence has found that activation of nuclear factor kappa-B (NF-κB), a major factor associated with inflammation in OA pathogenesis, induced hypoxia-inducible factor 2 alpha (HIF-2α) expression, thereby downregulating glutathione (GSH) production and inhibiting glutathione peroxidase 4 (GPX4) activity to promote ferroptosis [7]. In a study of OA mouse model, chondrocyte ferroptosis plays an important role in OA and can be inhibited by nuclear factor red lineage 2-related factor 2 (Nrf2) signaling activation to reduce OA symptoms [8]. These evidences suggest that ferroptosis can be a potential therapeutic target for OA treatment, which raises hope for overcoming the therapeutic difficulties of OA [5–8]. Although some studies on ferroptosis in OA have been reported, they are still at an initial stage. Thereby, it is necessary to further investigate the underlying mechanisms.

Mitochondria are involved in various important physiopathological processes in cells [9]. During the process of ferroptosis, the morphological changes that occur in mitochondria differ significantly from other forms of cell death, such as mitochondrial fragmentation, increased density, outer membrane rupture, and loss or disorganization of most of the mitochondrial cristae [10]. Intracellular mitochondrial homeostasis is maintained by the production of new mitochondria through mitochondrial biogenesis and the degradation of damaged mitochondria through mitophagy [11]. PTEN-induced putative kinase 1 (Pink1)-mediated Parkin-dependent mitophagy is the classical pathway for activating mitophagy [12]. Lin et al. [13] found that mitophagy reduces abnormal reactive oxygen species (ROS) production and alleviates oxidative stress, thus acting to reduce ferroptosis. However, the role of mitophagy in ferroptosis remains controversial. Li et al. [14] found that β-amyloid protein induces ferroptosis in Alzheimer’s disease dependent on Pink1/Parkin pathway mitophagy. In addition, the role of mitophagy in OA has been reported to be debated [15–17].Therefore, the relationship between them needs to be further investigated.

Peroxisome proliferator-activated receptor-γ (PPARγ) is a ligand-activated transcription factor. It regulates the expression of several genes and is essential for lipid and glucose metabolism [18]. PPARγ is now known to have antioxidant and antifibrotic effects in various pathophysiological processes [18, 19]. PPARγ deficiency leads to spontaneous osteoarthritis [20], and notably, PPARγ expression is reduced in human osteoarthritic cartilage compared with normal cartilage [21]. Past reports and our previous research have shown that PPARγ is a key regulator in maintaining cartilage health and has a chondroprotective role in osteoarthritis [22–24]. However, whether PPARγ protects cartilage associated with ferroptosis and its mechanism remains unclear.

In the current work, we aimed to explore the effect of PPARγ on ferroptosis in OA and its potential mechanisms. Overall, we demonstrate that PPARγ activation attenuates OA by inhibiting chondrocyte ferroptosis through promoting Pink1/Parkin-dependent mitophagy.

## Methods

### Animal experiment

Sprague–Dawley (SD) rats (male, 6–8 weeks) were randomly divided into the sham group, Hulth group, and pioglitazone group (*n* = 6). In the sham group, skin and joint capsules were incised and then sutured. The rat knee OA model was established using the modified Hulth method (Hulth group and pioglitazone group) [25]. A medial parapatellar incision was taken to open the right knee joint cavity of the rats, and the medial collateral ligament is resected. The knee is flexed, the anterior cruciate ligament is located and removed, and then, the medial meniscus is removed. Postoperatively, all animals moved freely in cages. The pioglitazone group was administered orally a dose of 20 mg/kg/d of pioglitazone (P816957, Macklin, Shanghai, China) suspended in 0.5% (w/v) methylcellulose (C104985, Aladdin, Shanghai, China) as described previously [26], and the sham and Hulth groups were fed with placebo (methylcellulose). Treatment started the day after surgery, and all animals were killed after 4 weeks.

### Macroscopic observation, histology, and immunohistochemistry

Rat knee joints were collected for macroscopic observation. Photographs of the macroscopic appearance were taken using a microscope (Nikon, SMZ18). Immersion of joint tissue in 4% paraformaldehyde (PFA, P0099, Beyotime) for 24 h. During 4 weeks, the joint tissues were decalcified in 10% EDTA (G1105, Servicebio). After decalcification, tissues were embedded using paraffin. Specimens were cut into slices for staining with Safranin O/fast green (SOFG, G1053, Servicebio). Slices were evaluated using the Osteoarthritis Research Society International (OARSI) scoring system [25].

*Immunohistochemical staining* The sections were deparaffinized, and the antigen was retrieved, blocked, and incubated overnight with a primary antibody COL2A1 (1:100; GB11021, Servicebio), MMP13 (1:100; GB11247, Servicebio), ACSL4 (1:100; 22401-1-AP, Proteintech, Wuhan, China), PPARγ (1:100; 16643-1-AP, Proteintech), Pink1 (1:50; 23274-1-AP, Proteintech), or Parkin (1:100; 14060-1-AP, Proteintech) in the refrigerator (4 °C). The next day, sections were incubated for 1 h with antibodies (GB23303, Servicebio), developed with a DAB solution (G1212-200T, Servicebio), and counterstained with hematoxylin (G1004, Servicebio). Immune-positive signals were observed under a microscope (Nikon, Eclipse Ci) and quantified using ImageJ 1.53t as described previously [27], and the data were expressed as integrated optical density (IOD).

### Isolation and culture of rat chondrocytes

Primary chondrocytes were extracted from 3-week-old SD rats. Cartilage was obtained from the knee joint and then cut into 2 mm^3^ pieces, and digested with 0.25% trypsin (25200072, Gibco, Grand Island, NY, USA) for 40 min. The pieces were digested with 0.3% collagenase II (C6885, Sigma, MO, USA) for 6 h at 37 °C after being rinsed three times with phosphate-buffered saline (PBS) (21-040-CVC, Corning, Shanghai, China), pH 7.4. Chondrocytes were obtained by centrifuging the cell suspension and resuspended in DMEM/F12 medium (C11330500BT, Gibco, Beijing, China) supplemented with 10% fetal bovine serum (10099141, Gibco, CA, USA) and 1% penicillin/streptomycin (15140122, Gibco, CA, USA). All cells were cultured in an incubator (5% CO_2_, 37 °C), with the medium changed every other day.

### Toluidine blue staining

To identify the chondrocytes, chondrocytes were seeded into a 12-well plate. After the cells were grown to 80% concentration, the medium was removed and washed 2 times with PBS. 4% PFA (Beyotime) fixed cells for 10 min, then PBS washed 3 times. Toluidine blue staining solution (G3660, Solarbio, Beijing, China) was added to the wells and stained for 5 min, and then, equal amount of distilled water was added and mixed, left for 15 min, washed twice with distilled water, and observed under the microscope.

### Alcian blue staining

Chondrocytes were seeded into a 12-well plate. After the cells were grown to 80% concentration, the medium was removed and washed 2 times with PBS. 4% PFA (Beyotime) fixed cells for 10 min, then PBS washed 3 times. Alcian blue staining solution (ALCB-10001, OriCell, Guangzhou, China) was added to the wells and stained for 60 min (37 °C). Remove the staining solution, wash 3 times with distilled water, and observe under the microscope.

### Cell viability assay

Chondrocytes were seeded into 96-well plates (4000/well) and incubated overnight in 100 µL/well of culture medium. For the experiment, the media was replaced with 100 µL of media containing different drugs. After RSL3 (SML2234, Sigma) or/and pioglitazone (PHR1632, Sigma) treatment, the medium was changed to a new medium (100 µL) containing 10 µL CCK-8 solution (C0038, Beyotime, Shanghai, China). Following 1 h of incubation, a microplate reader (GloMax Multi Plus, Promega, Sunnyvale, CA, USA) was used to measure the optical density (450 nm).

### Detection of intracellular reactive oxygen species (ROS) and lipid peroxidation (LPO)

Chondrocytes were seeded in 6-well plates (2 × 10^5^ cells/well) and incubated for 24 h. They were cultured in a medium containing 40 μmol of pioglitazone alone or combined with 10 μM GW9662 (HY-16578, MCE, NJ, USA), 10 μM Mdivi-1 (HY-15886, MCE), or 20 μM chloroquine (C843545, Macklin) for 24 h, followed by 4 h incubation with 0.2 μM RSL3. Intracellular ROS and LPO were detected using DCFH-DA (S0033S, Beyotime) and BODIPY 581/591 C11 (D3861, Invitrogen, CA, USA) fluorescent probes. The plates were washed with PBS, and then, 10 μM DCFH-DA or 5 μM BODIPY 581/591 C11 was added and placed in the incubator for 30 min. After washing twice with PBS, cells were collected and subjected to flow cytometry (BD FACSVerse, Becton Dickinson). FlowJo v10 (BD Bioscience) was used for data analysis.

### Measurement of mitochondrial membrane potential (MMP)

The mitochondrial membrane potential of chondrocytes was measured in 6-well plates by a JC-1 kit (C2006, Beyotime). After drug treatment, chondrocytes were washed twice with PBS and then cultured in JC-1 staining working solution for 30 min protected from light. The plates were washed twice using the JC-1 washing solution. Photographs were taken using a fluorescence microscope (Nikon, Eclipse-Ti) and analyzed using ImageJ software.

### Measurement of adenosine triphosphate (ATP) and malondialdehyde (MDA)

Enhanced ATP Assay Kit (S0027, Beyotime) and MDA Assay Kit (S0131S, Beyotime) were purchased from Beyotime. Chondrocytes in 6-well plates were lysed using cell lysis solution and then centrifuged to obtain the supernatant. Samples are tested according to the instructions using the appropriate working solution. The absorbance at the corresponding wavelength was measured using a spectrophotometer. The protein concentration measured by BCA Protein Concentration Assay Kit (P0012S, Beyotime) was used to normalize the ATP and MDA content.

### Measurement of intracellular iron

Iron Analysis Kit (MAK025, Sigma) purchased from Sigma was used to detect intracellular iron. Chondrocytes were planted in 6-well plates (2 × 10^5^ cells/well) for 24 h before giving drug treatment. Cells were washed once with PBS and collected after adding iron analysis solution. After centrifuging (16,000 g, 4 °C, 10 min), samples and iron-reducing agents were cultured and protected from light in 96-well plates (25 °C, 30 min). The iron ion probe (100 µL) was then added, and the plates were placed in darkness (25 °C, 1 h). A microplate reader (GloMax Multi Plus, Promega) was used to determine the optical density at 600 nm.

### Detection of mitochondrial superoxide (MitoSOX)

MitoSOX was measured using the MitoSOX Red dye (M36008, Invitrogen). Chondrocytes of 12-well plates were processed with the corresponding conditions. Cells were washed with PBS and incubated with MitoSOX Red dye (4 μM) at 37 °C for 10 min, followed by nuclei staining using Hoechst 33,258 (C1011, Beyotime) for 5 min. In the end, the plates were washed twice with PBS and captured in a fluorescence microscope. The fluorescence signal was quantified using ImageJ.

### Immunofluorescence staining

Chondrocytes were inoculated into 24-well plates. After cell treatment, 4% paraformaldehyde (Beyotime) was used to fix cells. To increase cell permeability, 0.2% Triton X-100 (P0096, Beyotime) was added to each well and treated for 20 min. Then, 2% bovine serum albumin (BSA, V900933, Sigma) in PBST was added to each well for 1 h. Next, cells were incubated with GPX4 antibody (1:100; 381,958, ZEN-BIOSCIENCE, Chengdu, China), or COL2A1 antibody (1:200; GB11021, Servicebio) dissolved in primary antibody dilution (P0023A, Beyotime) overnight (4 °C). The next day, Cy3-conjugated secondary antibody (1:100; GB21303, Servicebio, Wuhan, China) was added for 1 h. Finally, we used Hoecsht33258 (C1011, Beyotime) to label the nuclei (5 min). Pictures were photographed under a fluorescence microscope (Nikon, Eclipse Ci). Data were analyzed using ImageJ.

### RNA extraction and RT-qPCR reaction

Total RNA was extracted from chondrocytes using TRIzol reagent (15596026CN, Invitrogen). The cDNA was synthesized using PrimeScript RT Master Mix (RR036A, Takara, Beijing, China). RT-qPCR reaction reactions were performed with the TB Green RT-PCR reagent (RR420A, Takara). Relative mRNA expressions were normalized to GAPDH using the 2^−ΔΔCt^ method. These primer sequences are listed in Table 1.

**Table 1:** Primers information (5′–3′)

Gene	Forward primer 5′-3′	Reverse primer 5′-3′
PPARγ	TGAAGGCTCATATCTGTCTCCG	CATCGAGGACATCCAAGACAAC
ACSL4	CCCCAGACACACCGATTCAT	GAGCGCCAACTCTTCCAGTA
Ptgs2	ATGTTCGCATTCTTTGCCCAG	TACACCTCTCCACCGATGAC
GAPDH	GGGCTGGCATTGCTCTCAA	GTATCCTTGCTGGGCTGGG

### Western blot

Chondrocytes were lysed using RIPA Lysis Buffer (P0013B, Beyotime) containing a 1% protease inhibitor cocktail (P8340, Sigma). After electrophoresis, proteins were transferred to PVDF membranes (IPVH00010, Sigma) and incubated with primary antibodies in a refrigerator (4 °C) overnight. The next day, the PVDF membranes were washed three times with TBST and incubated with secondary antibodies (1:5000; AP132P, AP124P, Sigma) for 2 h and then visualized using the SuperSignal West Pico Chemiluminescent Substrate (34580, Thermo Scientific, CA, USA) in Bio-RAD ChemiDoc XRS + systems (Bio-Rad, CA, USA).

The primary antibodies: GAPDH antibody (1:1000; ARG10112, Arigo, Taiwan, China), PPARγ (1:2000; 16643-1-AP, Proteintech), GPX4 (1:1000; 381958, ZEN-BIOSCIENCE), Parkin (1:2000; 14060-1-AP, Proteintech), Pink1 (1:1000; 23274-1-AP, Proteintech), LC3B (1:1000; 18725-1-AP, Proteintech).

### Statistical analysis

All data were expressed as mean ± SD and analyzed with GraphPad Prism 8.0.2 (GraphPad Software). Statistical analysis was performed using one-way ANOVA followed by LSD test. *P* < 0.05 was considered statistically significant.

## Results

### Impaired PPARγ and chondrocyte ferroptosis are associated with osteoarthritis

**Fig. 1:**
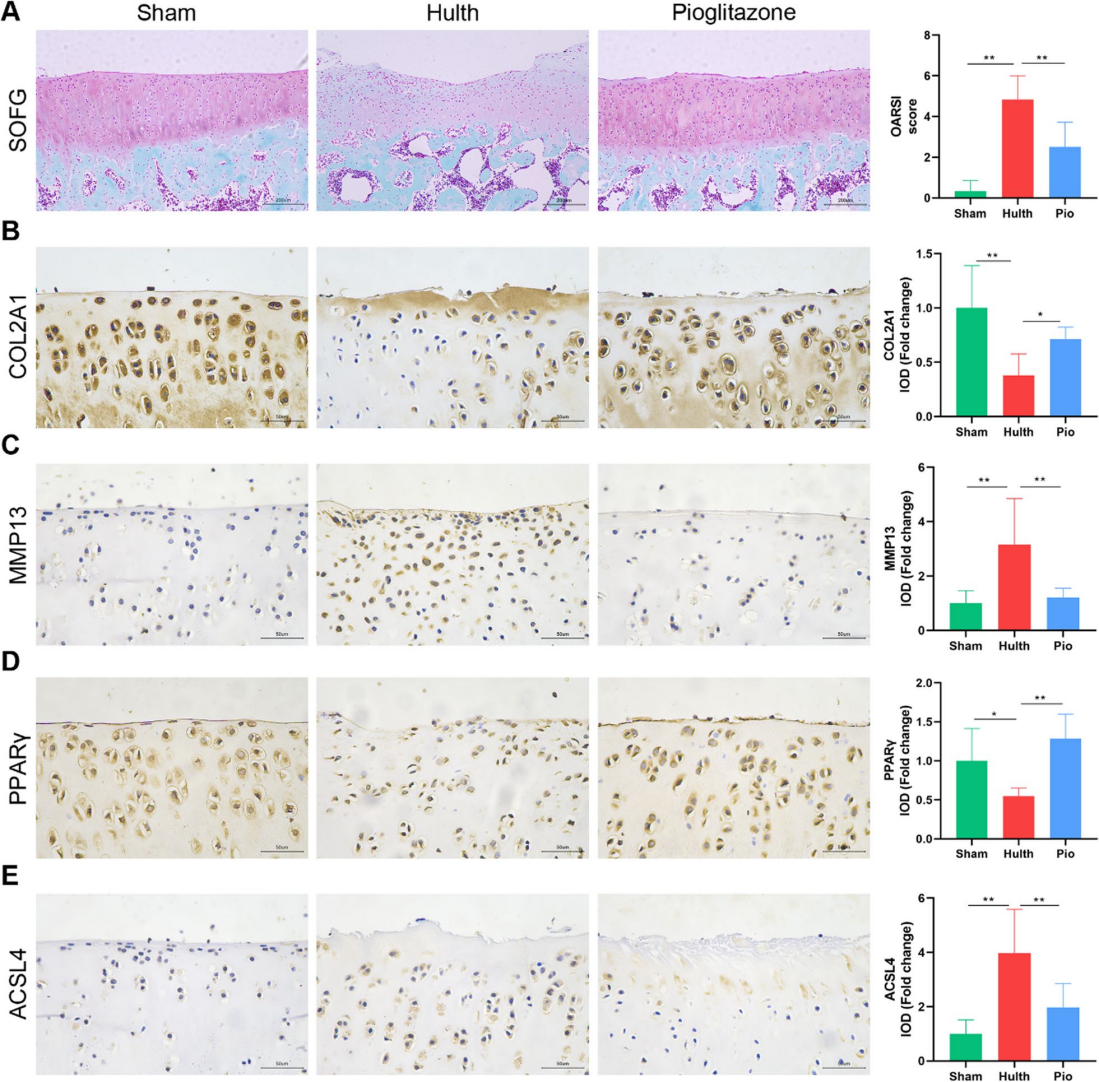
PPARγ activation attenuates osteoarthritis in rats, accompanied by inhibition of ferroptosis. **A** Cartilage sections were stained by Safranin O/fast green staining and evaluated with OARSI score (*n* = 6; Scale bars, 200 µm). **B**–**E** Representative pictures of COL2A1, MMP13, PPARγ, and ACSL4 immunohistochemical staining in rat cartilage samples. Quantitative analysis results are on the right (*n* = 6; Scale bars, 50 µm). Data were expressed as mean ± S.D. *p < 0.05; **p < 0.01

The macroscopic photographs of the rat knee joint showed that the cartilage of the sham group was shiny and smooth, with no obvious cartilage defects or osteophytes (Additional file 1: Fig. S1). The articular cartilage in the Hulth group was rough and lusterless, with obvious wear and erosion, exposed subchondral bone, and a large number of osteophytes (Additional file 1: Fig. S1). The articular cartilage of the pioglitazone group was slightly shiny, and the cartilage was smooth and slightly worn, with no obvious subchondral bone exposure, and few osteophytes (Additional file 1: Fig. S1). Safranin O/Fast Green staining was employed to examine the degradation in the cartilage, and we found smooth joint surfaces with clearly visible cartilage structures and abundant proteoglycan in the sham group (Fig. 1A). We observed significant cartilage degeneration as well as loss of proteoglycan in the Hulth group (Fig. 1A). The changes in the knee joints of pioglitazone-treated OA rats were significantly less pathological than those in the Hulth group, with smoother cartilage and richer proteoglycan content (Fig. 1A). OARSI scores were elevated in the Hulth group compared to the sham group and decreased in the pioglitazone (Pio)-treated group compared to the Hulth group (Fig. 1A). Meanwhile, we examined the osteoarthritis markers collagen type II (COL2A1) and matrix metalloproteinase 13 (MMP13). The Hulth model group had significant osteoarthritis manifestations, and the pioglitazone group improved the changes of these indexes, indicating that PPARγ agonists have the effect of attenuating osteoarthritis in rats (Fig. 1B, C). Moreover, PPARγ expression in rat cartilage was decreased in the Hulth group but recovered in the pioglitazone group (Fig. 1D). Finally, the expression of the ferroptosis marker acyl-CoA synthetase long-chain family member 4 (ACSL4) was elevated in the Hulth group and decreased after pioglitazone treatment (Fig. 1E). These findings suggest that PPARγ activation has a chondroprotective effect in osteoarthritis and may regulate chondrocyte ferroptosis.

### PPARγ activation attenuates RSL3-induced ferroptosis in rat chondrocytes

**Fig. 2:**
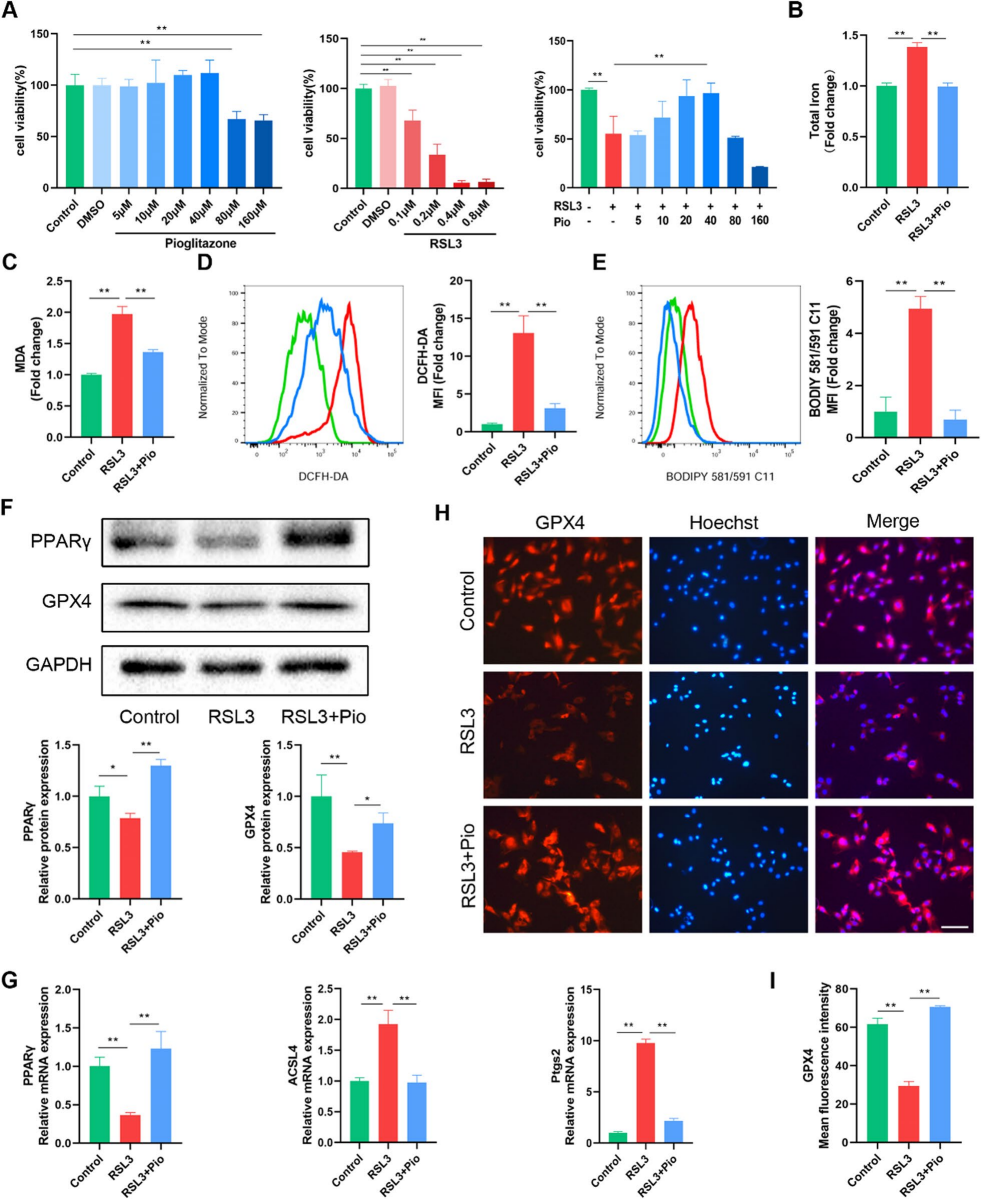
PPARγ activation attenuates RSL3-induced ferroptosis in rat chondrocytes. **A** Rat chondrocytes were treated for 24 h with pioglitazone (5, 10, 20, 40, 80, 160 μM), RSL3 (0.1, 0.2, 0.4, 0. 8 μM) for 4 h or pretreated with pioglitazone (5, 10, 20, 40, 80, 160 μM) for 24 h and then cultured for 4 h combine with RSL3 (0.2 μM). The CCK-8 test was used to determine cell viability (*n* = 3). **B**, **C** Intracellular total iron and MDA were measured using commercial kits, respectively (*n* = 3). **D**, **E** ROS and LPO were labeled with DCFH-DA and BODIPY 581/591 C11 dyes, respectively. Fluorescence signal detected by flow cytometry. The fold change of mean fluorescence intensity (MFI) was presented (*n* = 3). **F** The protein levels of PPARγ and GPX4 were determined by western blot (*n* = 3). **G** The mRNA PPARγ, ACSL4, and Ptgs2 expression levels were detected using RT-qPCR (*n* = 3). **H**, **I** Immunofluorescence staining of GPX4 in rat chondrocytes (*n* = 3; Scale bars, 100 µm). Data were expressed as mean ± S.D. *p < 0.05; **p < 0.01

Primary chondrocytes were identified using toluidine blue, Alcian blue, and type II collagen immunofluorescence staining (Additional file 1: Fig. S2). We used the ferroptosis inducer RSL3 to establish a ferroptosis model in chondrocytes cultured in vitro. The toxicity of pioglitazone and RSL3 on chondrocytes was examined by the CCK-8 method. Chondrocytes treated with 40 µM pioglitazone or less for 24 h showed no significant cytotoxicity, whereas RSL3 reduced cell viability for 4 h in a concentration-dependent manner (Fig. 2A). Considering that 0.2 µM RSL3 reduces cellular viability by approximately 50–60% at 4 h, we chose this dose and time for the study. After pretreatment with varied doses of pioglitazone for 24 h, chondrocytes were incubated for 4 h with 0.2 M RSL3. We found that 40 µM pioglitazone significantly protected chondrocytes from the RSL3-induced reduction in cell viability (Fig. 2A). In addition, we examined the changes in cellular PPARγ expression. PPARγ expression was significantly lower in the RSL3 group compared to the control group, according to Western blot and RT-qPCR data, and pioglitazone restored PPARγ expression (Fig. 2F, G).

To further examine the changes in chondrocyte ferroptosis, we measured several indicators associated with ferroptosis. RSL3 treatment of chondrocytes resulted in a significantly greater intracellular iron content compared to the control group, while the excess intracellular iron content was reduced after pioglitazone treatment (Fig. 2B). Meanwhile, intracellular MDA, which is a lipid peroxidation end product, was increased in the RSL3 group, and pioglitazone inhibited the intracellular MDA changes caused by RSL3 (Fig. 2C). In the process of ferroptosis, the accumulation of ROS mediates the key step of lipid peroxidation. Therefore, we detected intracellular ROS and LPO using fluorescent probes with DCFH-DA and BODIPY 581/591 C11. Flow cytometry results showed that intracellular ROS and LPO were significantly higher in the RSL3 group and lower in the pioglitazone group (Fig. 2D, E). Another feature of ferroptosis is the reduced expression of the antioxidant enzyme GPX4. Therefore, we examined changes in GPX4. Western bolt and cellular immunofluorescence results showed that RSL3 significantly decreased GPX4 expression, and this change was restored by pioglitazone (Fig. 2F, H, I). Finally, we also examined the additional ferroptosis markers ACSL4 and prostaglandin-endoperoxide synthase 2 (Ptgs2). The mRNA expression of ACSL4 and Ptgs2 was increased in the RSL3 group compared to the control group, according to RT-qPCR data, and pioglitazone significantly reduced the elevated ACSL4 and Ptgs2 (Fig. 2G). Taken together, we confirmed that PPARγ activation in chondrocytes inhibits RSL3-induced ferroptosis.

### PPARγ activation attenuates chondrocyte mitochondrial damage and restores impaired mitophagy

**Fig. 3:**
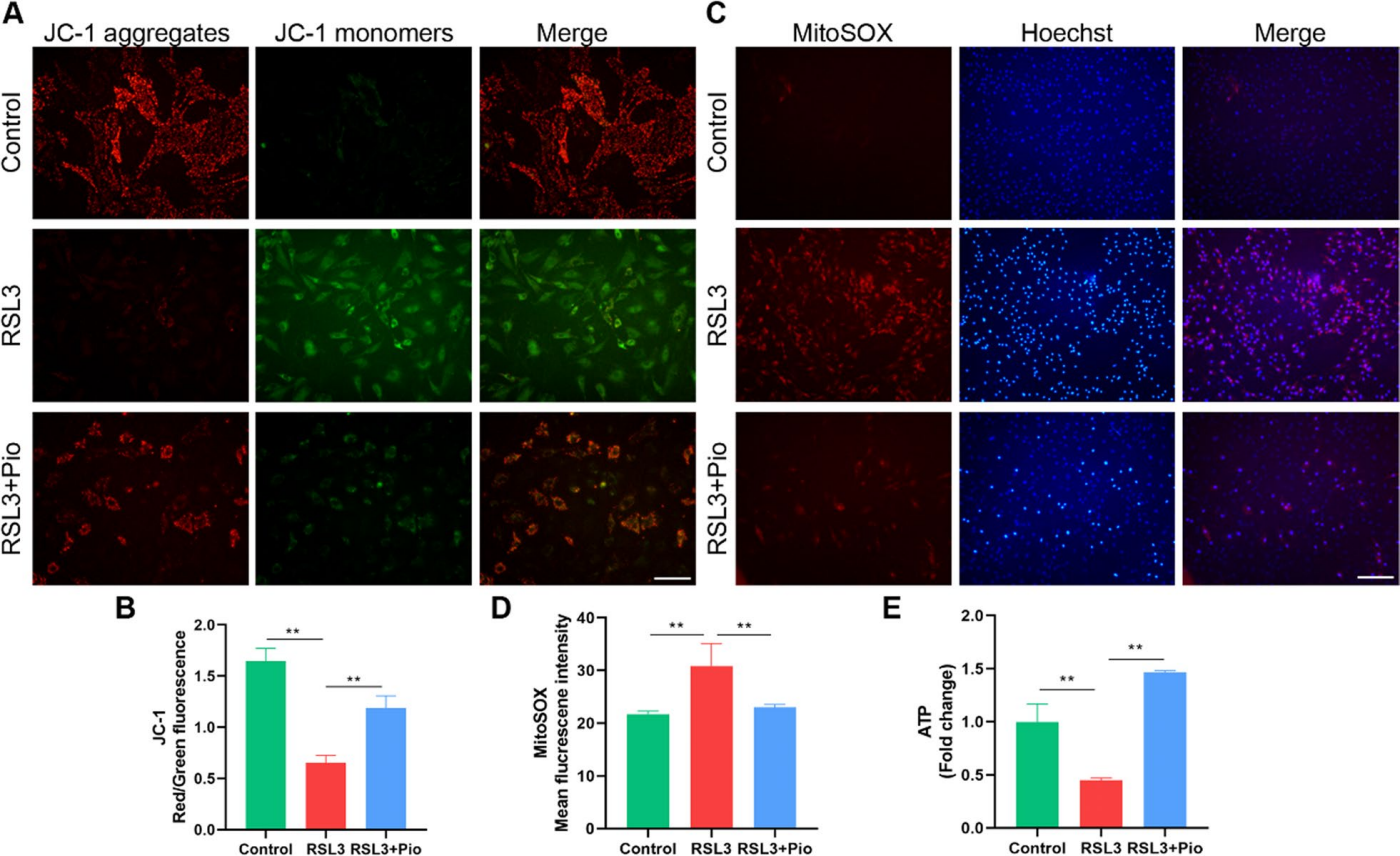
PPARγ activation attenuates chondrocyte mitochondrial damage caused by ferroptosis. **A**, **B** JC-1 images and the ratio of red/green fluorescence in chondrocytes (*n* = 3, Scale bars, 100 µm). **C**, **D** MitoSOX staining was used to detect mtROS levels. Mean fluorescence intensities were used for statistical analysis (*n* = 3, Scale bars, 200 µm). **E** ATP production was measured using commercial kits (*n* = 3). Data were expressed as mean ± S.D. *p < 0.05; **p < 0.01

Mitochondria are engaged in a variety of cell death, including mediating ferroptosis signals, and mitochondria undergo morphological changes that distinguish ferroptosis from other types of cell death. JC-1 staining of mitochondrial membrane potential (MMP) is one of the commonly used indicators of mitochondrial function. We found that red fluorescence was attenuated and green fluorescence was enhanced in the RSL3 group, as well as the ratio of red to green fluorescence signal dropped. By contrast, the altered mitochondrial membrane potential was restored in the pioglitazone group (Fig. 3A, B). We also examined mitochondrial ROS (mtROS), and as expected, mtROS was significantly increased in RSL3-treated chondrocytes, while pioglitazone treatment inhibited mtROS production (Fig. 3C, D). Mitochondria are the energy centers of cells, and ATP production is an important indicator of mitochondrial function, so we examined ATP production. RSL3 treatment significantly reduced chondrocyte ATP generation compared to the control group, but pioglitazone treatment boosted cellular ATP production compared to the RSL3 group (Fig. 3E). Our data suggest that PPARγ activation can attenuate mitochondrial damage in chondrocytes caused by ferroptosis.

**Fig. 4:**
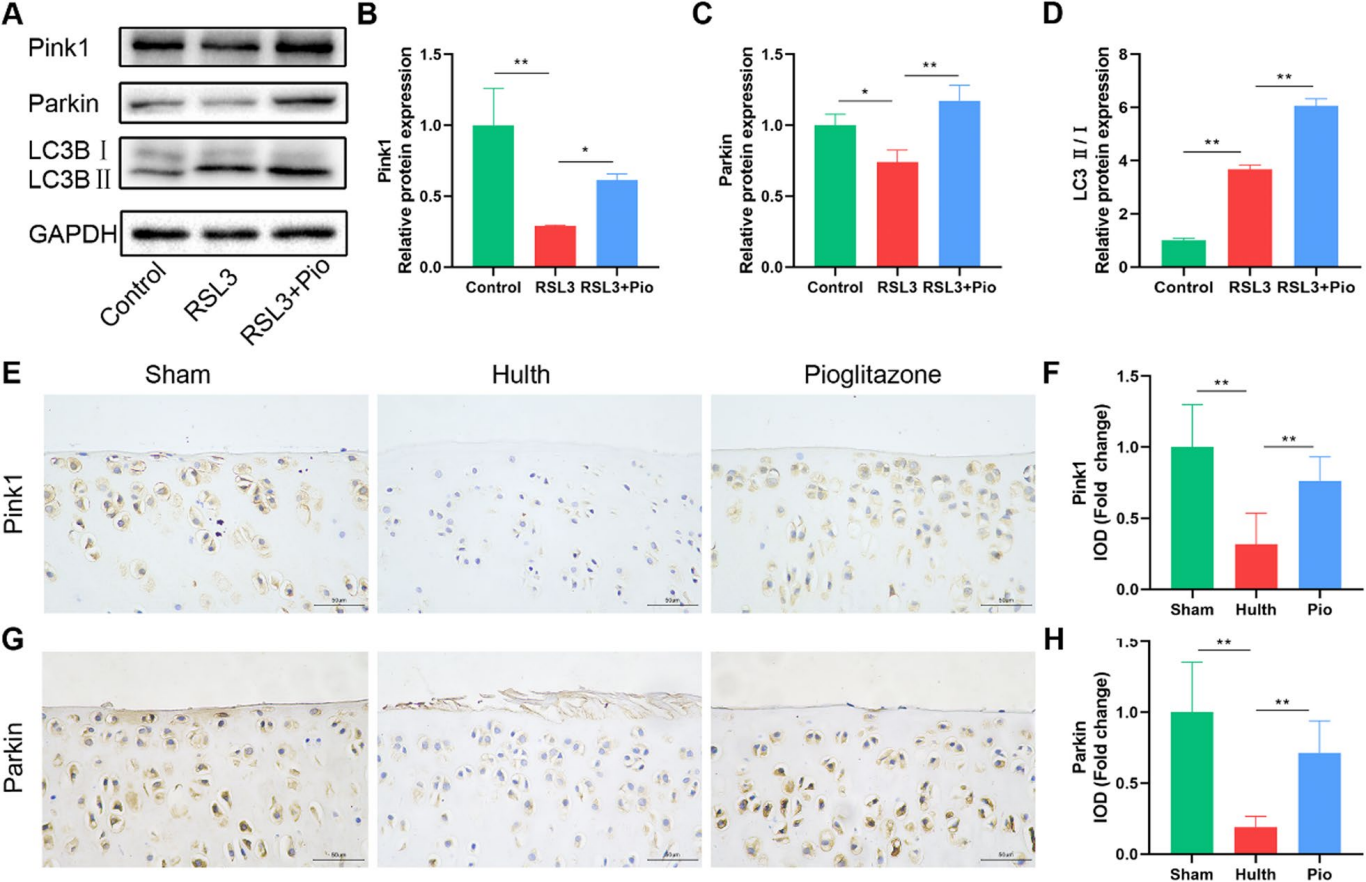
PPARγ activation restores impaired mitophagy. **A**–**D** Pink1, Parkin, and LC3B, with GAPDH as an internal control detected by western blot. Statistical analysis of western blotting is located on the right (*n* = 3). **E**–**H** Representative pictures of Pink1 and Parkin immunohistochemical staining in rat cartilage samples. Quantitative analysis results are on the right (*n* = 6, Scale bars, 50 µm). Data were expressed as mean ± S.D. *p < 0.05; **p < 0.01

The role of mitophagy is to remove damaged mitochondria and maintain mitochondrial homeostasis. To study mitophagy in ferroptosis, we examined the mitophagy initiators Pink1 and Parkin. We found that RSL3 decreased Pink1 and Parkin expression and was restored by pioglitazone treatment (Fig. 4A–C). Microtubule-associated protein B-light chain 3 (MAP1LC3, LC3) is an indicator of autophagic flux. The ratio of LC3B-II/I in chondrocytes was elevated after treatment with RSL3, and we then find that it raised further in the pioglitazone group (Fig. 4A, D). In conclusion, these phenomena suggest that Pink1/Parkin-dependent mitophagy is blocked when chondrocyte ferroptosis occurs, and this alteration was restored after PPARγ activation. Consistent with this, immunohistochemical detection of Pink1 and Parkin in rat sections also showed the same trend (Fig. 4E–H). These findings suggest that PPARγ activation restores impaired mitophagy in chondrocytes.

### PPARγ mitigates chondrocyte mitochondrial damage through mitophagy

**Fig. 5:**
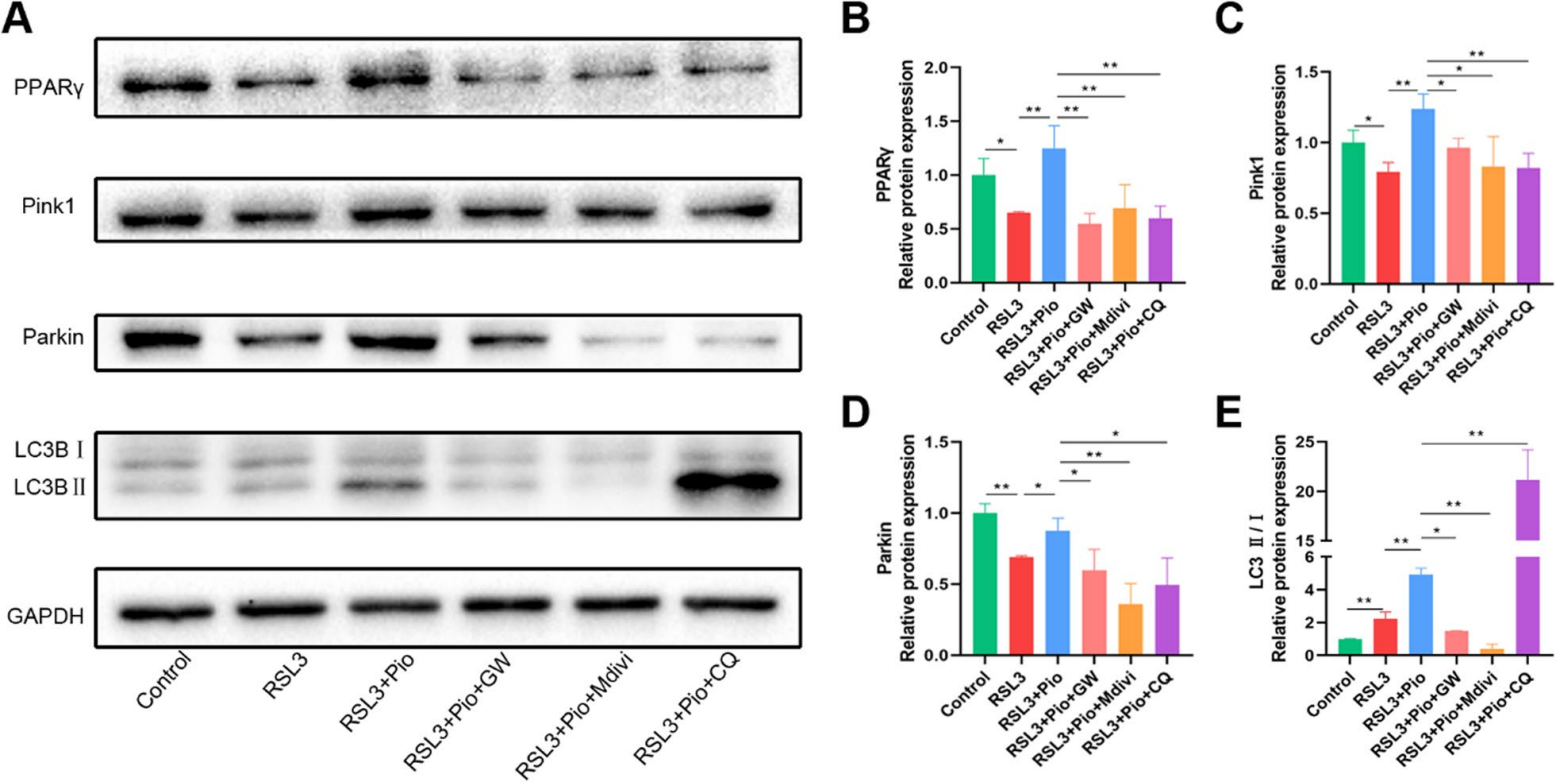
PPARγ activates Pink1/Parkin-dependent mitophagy in chondrocytes. **A**–**E** PPARγ, Pink1, Parkin, and LC3B, with GAPDH as an internal control detected by western blot. Statistical analysis of western blotting is located on the right (*n* = 3). Data were expressed as means ± S.D. *p < 0.05; **p < 0.01

Next, we investigated the potential mechanisms by which PPARγ attenuates mitochondrial dysfunction. Therefore, we treated cells with GW9662 (GW, PPARγ antagonist), and in addition, to investigate whether this process is associated with mitophagy, we applied Mdivi-1 (Mdivi, Mitophagy inhibitor) and chloroquine (CQ), a standard drug that inhibits mitophagy by blocking autophagosome–lysosome fusion at the final step of autophagy. Western blot showed that GW9662 significantly reduced PPARγ, Pink1, and Parkin expression (Fig. 5A–D), as well as the ratio of LC3B-II/I (Fig. 5A, E), and the same trend was obtained after the application of Mdivi-1 (Fig. 5A–E). After chloroquine inhibited mitochondrial autophagic flux, LC3B-II accumulated, LC3B-II/I elevated, and the levels of Pink1 and Parkin significantly reduced (Fig. 5A, C–E). The study suggests that PPARγ activates Pink1/Parkin-dependent mitophagy in chondrocytes.

**Fig. 6:**
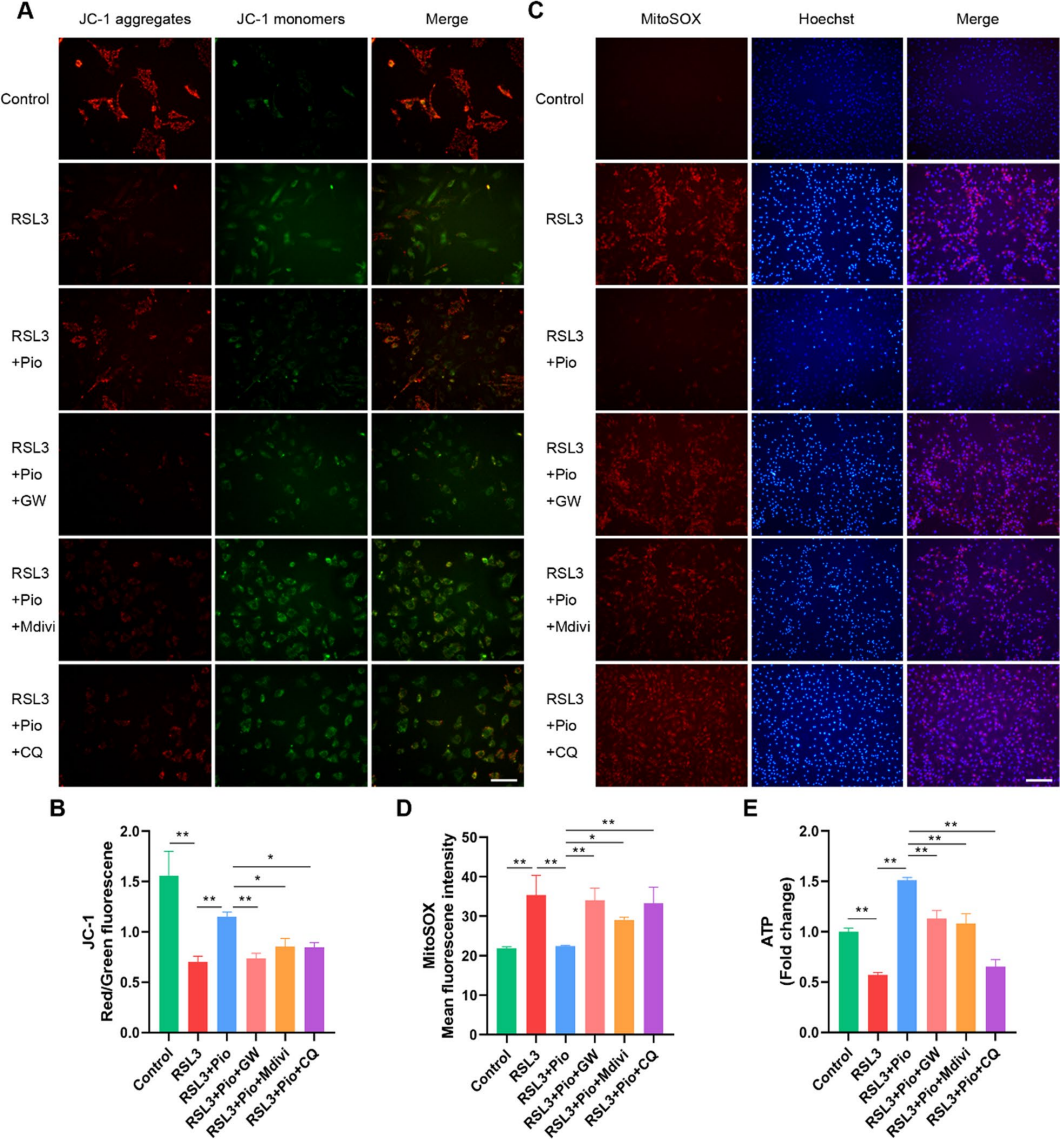
PPARγ mitigates chondrocyte mitochondrial damage through mitophagy. **A**, **B** JC-1 images and the ratio of red/green fluorescence in chondrocytes (*n* = 3; Scale bars, 100 µm). **C**, **D** MitoSOX staining was used to detect mtROS levels. Mean fluorescence intensities were used for statistical analysis (*n* = 3; Scale bars, 200 µm). **E** ATP production was measured using commercial kits (*n* = 3). Data were expressed as means ± S.D. *p < 0.05; **p < 0.01

We then examined the changes in mitochondrial function. JC-1 staining showed that blocking PPARγ or mitophagy significantly enhanced green fluorescence, significantly decreased red fluorescence, and decreased the red/green fluorescence ratio in comparison with the pioglitazone group (Fig. 6A, B). MitoSOX staining indicated that after inhibiting the aforementioned targets, the fluorescence intensity of mtROS was elevated than in the pioglitazone group (Fig. 6C, D). As expected, GW9662, Mdivi-1, and CQ all significantly reversed the promotion of cellular ATP production by pioglitazone (Fig. 6E). In conclusion, these results suggest that PPARγ attenuates mitochondrial damage through Pink1/Parkin-dependent mitophagy.

### Inhibition of PPARγ-mediated mitophagy aggravates chondrocyte ferroptosis

**Fig. 7:**
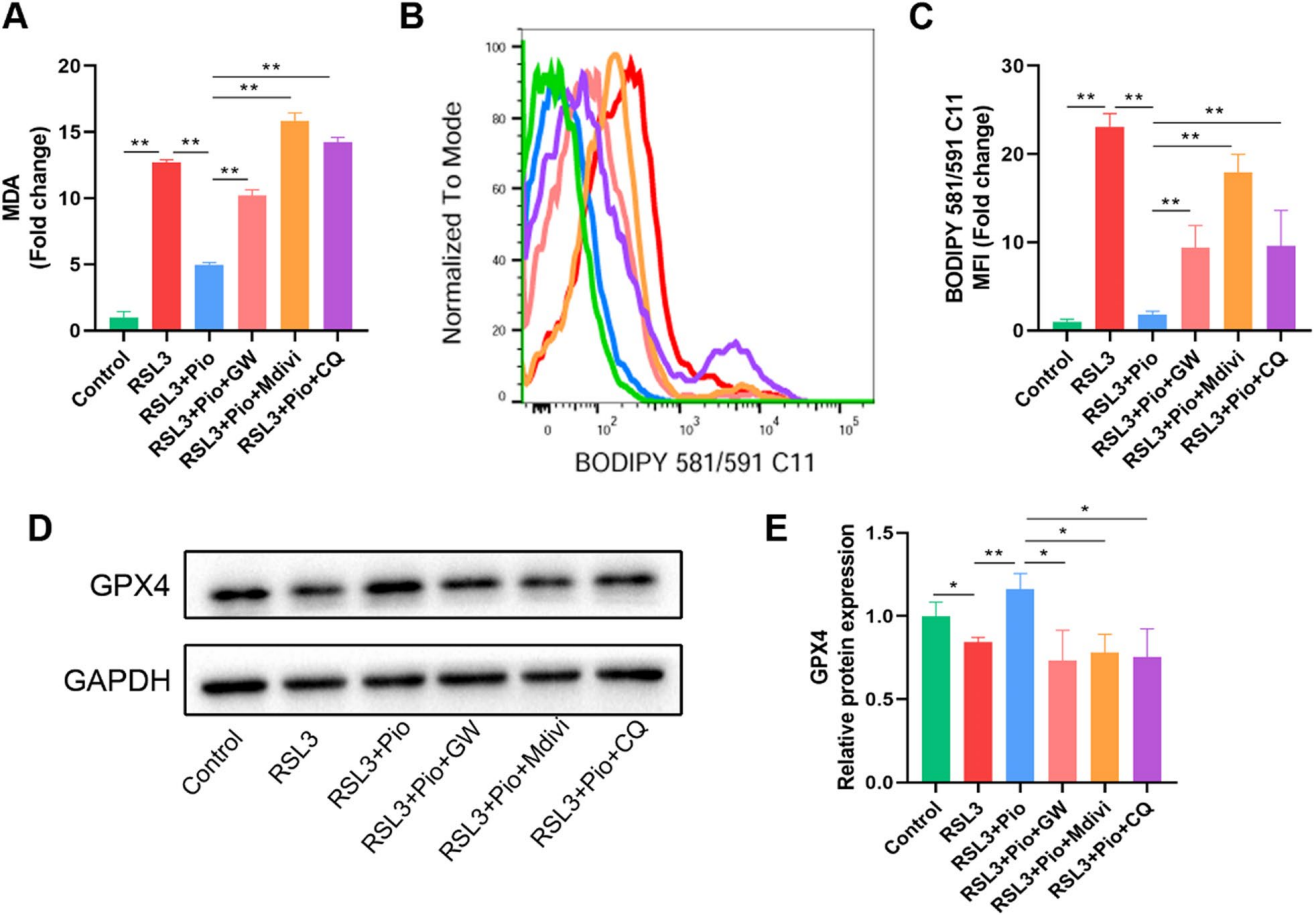
Inhibition of PPARγ-mediated mitophagy aggravates chondrocyte ferroptosis. **A** MDA were measured using commercial kits (*n* = 3). **B**–**C** LPO is labeled with BODIPY 581/591 C11 dye and assayed by flow cytometry. The fold change of MFI was presented (*n* = 3). **D**, **E** Western blot analysis of GPX4, with GAPDH as an internal control. Statistical analysis of western blotting is located on the right (*n* = 3). Data were expressed as means ± S.D. *p < 0.05; **p < 0.01

Lastly, we examined whether the PPARγ-mediated mitophagy in chondrocytes was associated with ferroptosis. We examined intracellular MDA, and the measurements showed that inhibition of PPARγ, or mitophagy elevated MDA (Fig. 7A). LPO detected by flow cytometry showed the same trend (Fig. 7B, C). In addition, we examined GPX4, a key indicator of ferroptosis, and western blot results showed that GW9662, Mdivi-1, and CQ inhibited the restorative effect of pioglitazone on GPX4 expression levels (Fig. 7D, E). These results indicate that PPARγ activation inhibits ferroptosis in chondrocytes through the Pink1/Parkin-dependent mitophagy pathway.

## Discussion

**Fig. 8:**
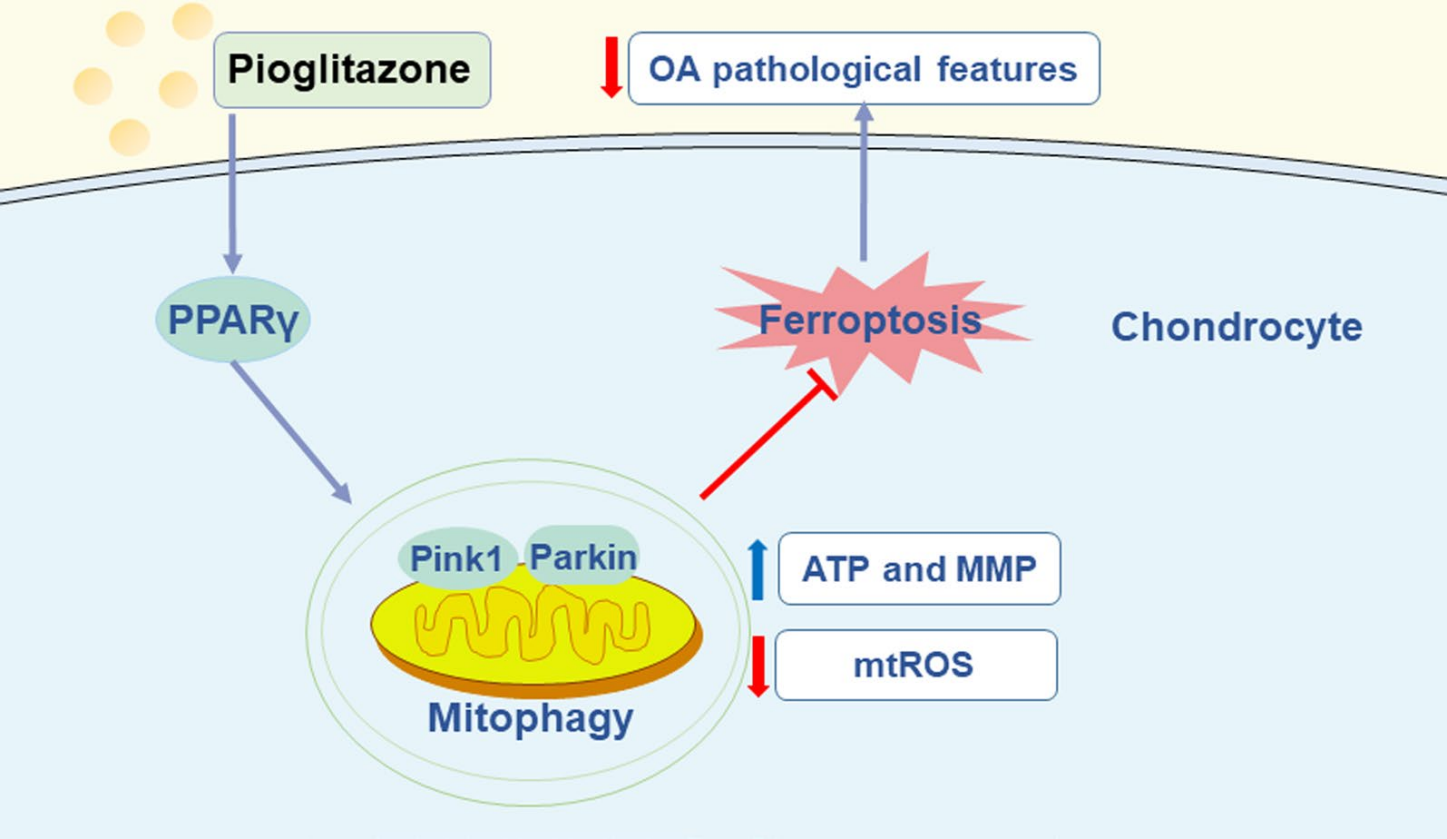
Schematic diagram of the potential mechanisms by which PPARγ activation suppresses chondrocyte ferroptosis through mitophagy in osteoarthritis. Activation of PPARγ by pioglitazone promotes the upregulation of Pink1 and Parkin, decreasing mtROS production, and restoring mitochondrial function, thereby suppressing the level of lipid peroxidation and ultimately inhibiting chondrocyte ferroptosis, alleviating OA symptoms

OA is a prevalent disease that afflicts the elderly and causes joint pain and dysfunction. However, current treatment and management strategies are unable to cure OA. Therefore, it is urgent to develop more effective treatments for OA. Progressive destruction of articular cartilage is characteristic of OA, and the maintenance of tissue function of articular cartilage is dependent on its sole resident cell type, the chondrocyte. Therefore, the death of chondrocytes is crucial in the pathogenesis of OA [28, 29]. Ferroptosis has recently been demonstrated to be associated with OA, and the blockage of chondrocyte ferroptosis has shown promise for the treatment of OA [5]. Although PPARγ has been shown to have chondroprotective effects [22], it is unclear whether PPARγ regulates chondrocyte ferroptosis. In this study, we demonstrates that the mechanism of the chondroprotective effect of PPARγ is mediated through the Pink1/Parkin-dependent mitophagy, thus inhibiting chondrocyte ferroptosis in OA (Fig. 8).

Chondrocytes are unique cells in the articular cartilage that regulate the balance of extracellular matrix synthesis and degradation and play a vital role in articular cartilage function [5, 28, 29]. Oxidative stress-induced chondrocyte death is a critical event during the development of OA, and existing researchers have identified apoptosis and necrosis as forms of chondrocyte death [28]. Recently, a study by Yao and coworkers demonstrated for the first time that chondrocytes underwent ferroptosis during inflammation and iron overload, inhibiting type II collagen expression and promoting MMP13 elevation, revealing the important role of ferroptosis in OA. This also revealed that inhibition of chondrocyte ferroptosis could reduce the manifestation of OA in vitro and in vivo, which provides a novel and effective way to treat OA [5]. Recently, a study by Miao and coworkers found that chondrocyte ferroptosis is also present in human OA [6]. We found that chondrocyte ferroptosis was increased in a surgically induced rat OA model and was closely associated with the manifestation of OA. It is suggested that chondrocyte ferroptosis is indeed involved in the progression of osteoarthritis and shows potential as a novel therapeutic target that seems to offer hope for overcoming the therapeutic challenges of OA.

In studies of OA, it has been shown that PPARγ expression is significantly reduced in human osteoarthritic cartilage compared with normal cartilage [21]. We found a significant inhibition of PPARγ expression in OA rats, which is consistent with previous studies [30]. However, scholars have conflicting views on the targets and pathways of PPARγ in osteoarthritis, with some studies demonstrating that PPARγ can maintain chondrocyte viability by the Akt/mTOR pathway to induce chondrocyte autophagy [31], and others finding that PPARγ can inhibit chondrocyte apoptosis and reduce the inflammatory response by inhibiting MAPK and NF-κB activation [32]. In addition, PPARγ has been reported to activate the AMPK-SIRT-1 pathway in human articular cartilage and attenuate inflammation [33]. However, our study found that PPARγ activation attenuated OA, and this effect was accompanied by a reduction in chondrocyte ferroptosis. Therefore, we hypothesized that PPARγ might attenuate OA in a manner that regulates chondrocyte ferroptosis.

According to the above-mentioned findings, we further studied the mechanism of PPARγ. In the RSL3-induced chondrocyte ferroptosis model, we found that PPARγ inhibited chondrocyte ferroptosis. First, we examined intracellular ROS, MDA, and LPO levels to understand the fluctuation of oxidative stress. Second, we assessed the cellular iron and the level of GPX4, a core protein of ferroptosis. We found that PPARγ reversed the alterations caused by the ferroptosis inducer RSL3. Moreover, we observed that RSL3 treatment reduced Pink1 and Parkin in chondrocytes and upgraded the ratio of LC3B-II/I, suggesting that mitophagy was blocked during ferroptosis in chondrocytes, which is supported by a study by Li and coworkers [34]. We discovered that PPARγ restored the reduction in Pink1 and Parkin expression caused by the ferroptosis inducer RSL3, and these phenomena suggest that PPARγ has a role in promoting mitophagy. This was also illustrated by functional assays of mitochondria, where PPARγ reduced the mtROS production in mitochondria caused by ferroptosis, reduced mitochondrial oxidative stress and interruption in chondrocytes, and restored cellular ATP generation and MMP. Application of GW9662, a classical specific antagonist of PPARγ, eliminated the effects of inhibiting chondrocyte ferroptosis and promoting mitophagy, demonstrating that these effects are PPARγ-dependent.

The studies on PPARγ-regulated Pink1/Parkin-dependent mitophagy are few and with mixed findings. PPARγ negatively regulates mitotophagy which has been reported in several studies. Small et al. [35] published a study in 2014 in which PPARγ agonists exhibited a pro-apoptotic effect in renal cells. In this study, aberrant PPARγ activation was found and Parkin-dependent mitophagy was activated at 2 h; however, it was inhibited at 18 h [35]. In another study of ischemia–reperfusion in renal tubular epithelial cells, Wei et al. [36] found that ischemia–reperfusion injury inhibited PPARγ expression and increased the expression of LC3II and Pink1, and that PPARγ was negatively correlated with LC3II and Pink1; however, the mechanism between PPARγ and Pink1-dependent mitophagy in this study was not clear. However, a recent study reported that PPARγ can promote mitophagy in the Pink1/Parkin pathway. Li et al. [37] published their insights into PPARγ regulation of Pink1/Parkin pathway mitophagy in Alzheimer’s disease. This study applied a novel PPARγ agonist, ligustrazine piperazine derivative, to treat Alzheimer’s disease and found that PPARγ activation reduced Aβ40 and Aβ42 levels in vivo and in vitro and improved cognitive impairment in mice [37]. The mechanism of this effect was demonstrated by promoting mitophagy in the Pink1/Parkin pathway, and this protective effect was abolished by the PPARγ-specific antagonist GW9662 [37]. We also found that PPARγ promotes the Pink1/Parkin pathway in the present study; however, the regulation of the PPARγ on the Pink1/Parkin pathway varies in different tissues, which needs to be further explored in the future.

Mitochondria are not only the primary location of intracellular ROS generation but are also involved in the regulation of redox associated with cell survival [10]. Studies have found a significant increase in mtROS during ferroptosis [38, 39], and the application of drugs targeting mitochondria attenuates ferroptosis after inhibition of mtROS production [39–41]. In various states of stress or oxidative damage, mitochondrial damage leads to abnormally high ROS, and mitophagy can scavenge damaged mitochondria and thus maintain redox homeostasis. Up to now, scholars still pay little attention to the relationship between mitophagy and ferroptosis. Lin et al. [13] found that activation of mitophagy can inhibit ferroptosis. However, Wang et al. [42] found that activation of mitophagy in osteoblasts leads to increased ferroptosis, and Basit et al. [43] found that inhibition of mitochondrial complex I stimulated mitophagy through mtROS production, and activation of mitophagy further promoted elevated mtROS, leading to necrosis and ferroptosis. Therefore, to clarify the role played by mitophagy in chondrocyte ferroptosis, we inhibited mitochondrial autophagic degradation using the mitophagy inhibitor Mdivi-1 and the classical autophagy blocker chloroquine. We found that after blocking mitophagy, mtROS was elevated, and ferroptosis was significantly increased, as evidenced by decreased GPX4 expression and increased MDA and LPO levels. Our study illustrates that the inhibition of chondrocyte ferroptosis by PPARγ is achieved through the mitophagy pathway, confirming the relationship between mitophagy and chondrocyte ferroptosis, which provides direct evidence for the anti-ferroptosis effect of mitophagy.

Oxidative stress leads to mitochondrial damage and dysfunction, and damaged mitochondria produce abnormally increased ROS leading to further aggravation of oxidative stress [44]. Therefore, mitochondrial homeostasis has an important role in maintaining normal cellular function. Mitophagy, a specific form of autophagy, is important for maintaining mitochondrial homeostasis by promoting the clearance of damaged mitochondria via the autophagic pathway [45]. Yet, there is disagreement on the significance of mitophagy in OA. Kim et al. [46] found that BNIP3-dependent mitophagy promotes cartilage degeneration. In addition, Shin et al. [17] reported that mitophagy proteins Pink1 and Parkin were expressed elevated in cartilage of human and monosodium iodoacetate-induced OA rat models and caused cartilage degeneration. Increasingly, a growing number of studies have confirmed the potential benefits of mitophagy in OA [15, 47–49]. In the current work, we found that the expression of Pink1 and Parkin reduced in rat OA, but PPARγ agonist restored these alterations. Based on our findings, we demonstrated the link between PPARγ and Pink1-dependent mitophagy, and the benefits of promoting mitophagy for chondrocytes.

## Conclusions

In summary, we demonstrated that PPARγ activation inhibits chondrocyte ferroptosis in osteoarthritis and that this chondroprotective effect is associated with the activation of Pink1/Parkin-dependent mitophagy. As a rescue mechanism to alleviate osteoarthritis, the chondroprotective effect of PPARγ was elucidated in the present study. Therefore, inhibition of chondrocyte ferroptosis by PPARγ may be a therapeutic approach for osteoarthritis.

### Supplementary Information

**Additional file 1**. Macroscopic appearance of rat knee joints; Identification of primary rat chondrocytes.

## Data Availability

The data presented in this study are available from the corresponding author upon reasonable request.

## Abbreviations

OA: Osteoarthritis
PPARγ: Peroxisome proliferator-activated receptor-γ
Pink1: PTEN-induced putative kinase 1
ACSL4: Acyl-CoA synthetase long-chain family member 4
HIF-2α: Hypoxia-inducible factor 2 alpha
GSH: Glutathione
GPX4: Glutathione peroxidase 4
Nrf2: Nuclear factor red lineage 2-related factor 2
ROS: Reactive oxygen species
SD: Sprague–Dawley
LPO: Lipid peroxidation
MMP: Mitochondrial membrane potential
ATP: Adenosine triphosphate
MDA: Malondialdehyde
MitoSOX: Mitochondrial superoxide
SOFG: Safranin O/fast green
OARSI: Osteoarthritis Research Society International
PFA: Paraformaldehyde
COL2A1: Collagen type II
MMP13: Matrix metalloproteinase 13
Ptgs2: Prostaglandin-endoperoxide synthase 2
mtROS: Mitochondrial ROS
GW: GW9662
Mdivi: Mdivi-1
CQ: Chloroquine
Pio: Pioglitazone
