# Supplementary material for: PPARγ activation suppresses chondrocyte ferroptosis through mitophagy in osteoarthritis
Source: J Orthop Surg Res. 2023 Aug 24;18:620. doi: 10.1186/s13018-023-04092-x (PMC10463860; doi:10.1186/s13018-023-04092-x)
Supplement: Supplementary file 1 — Additional file 1. Macroscopic appearance of rat knee joints; Identification of primary rat chondrocytes. [file 13018_2023_4092_MOESM1_ESM.docx]

Additional file 1

1. Figure S1


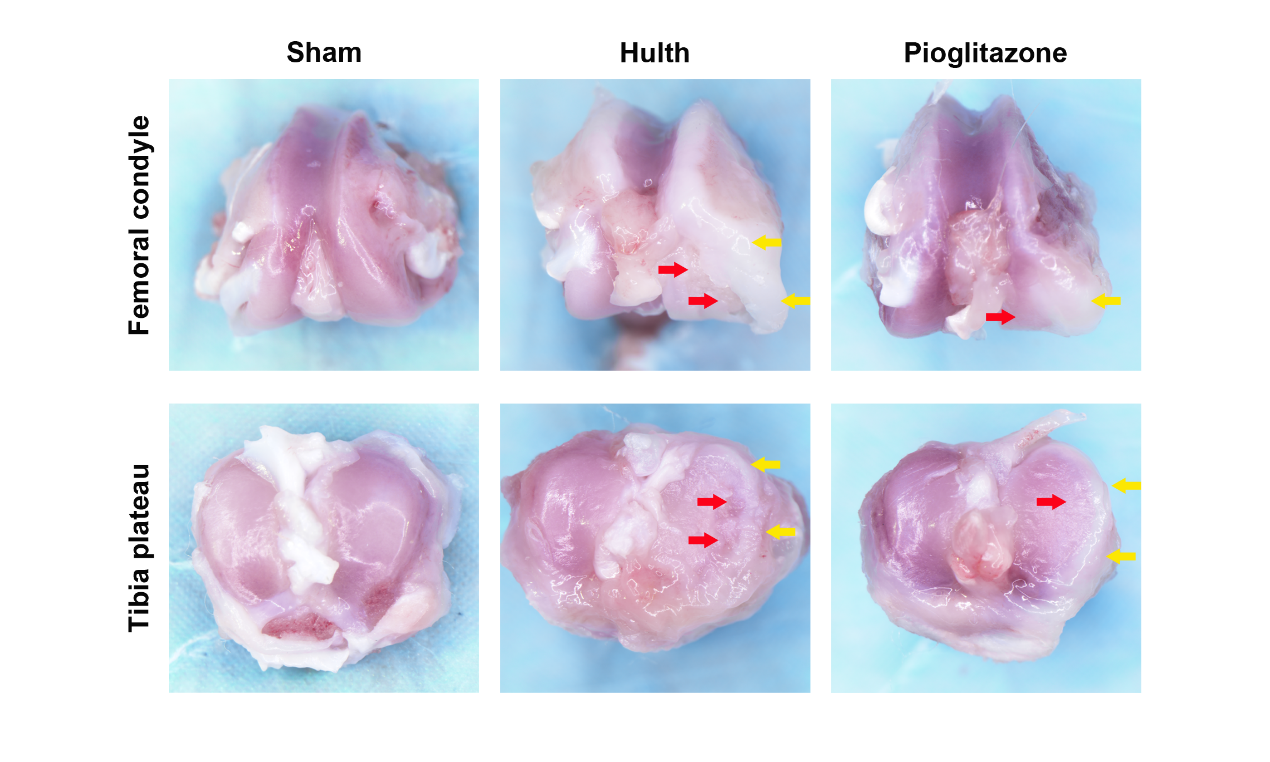


**Figure S1.** Representative macroscopic appearance of rat knee joints. Red arrows： wear of cartilage; yellow arrows: osteophytes.

2. Figure S2


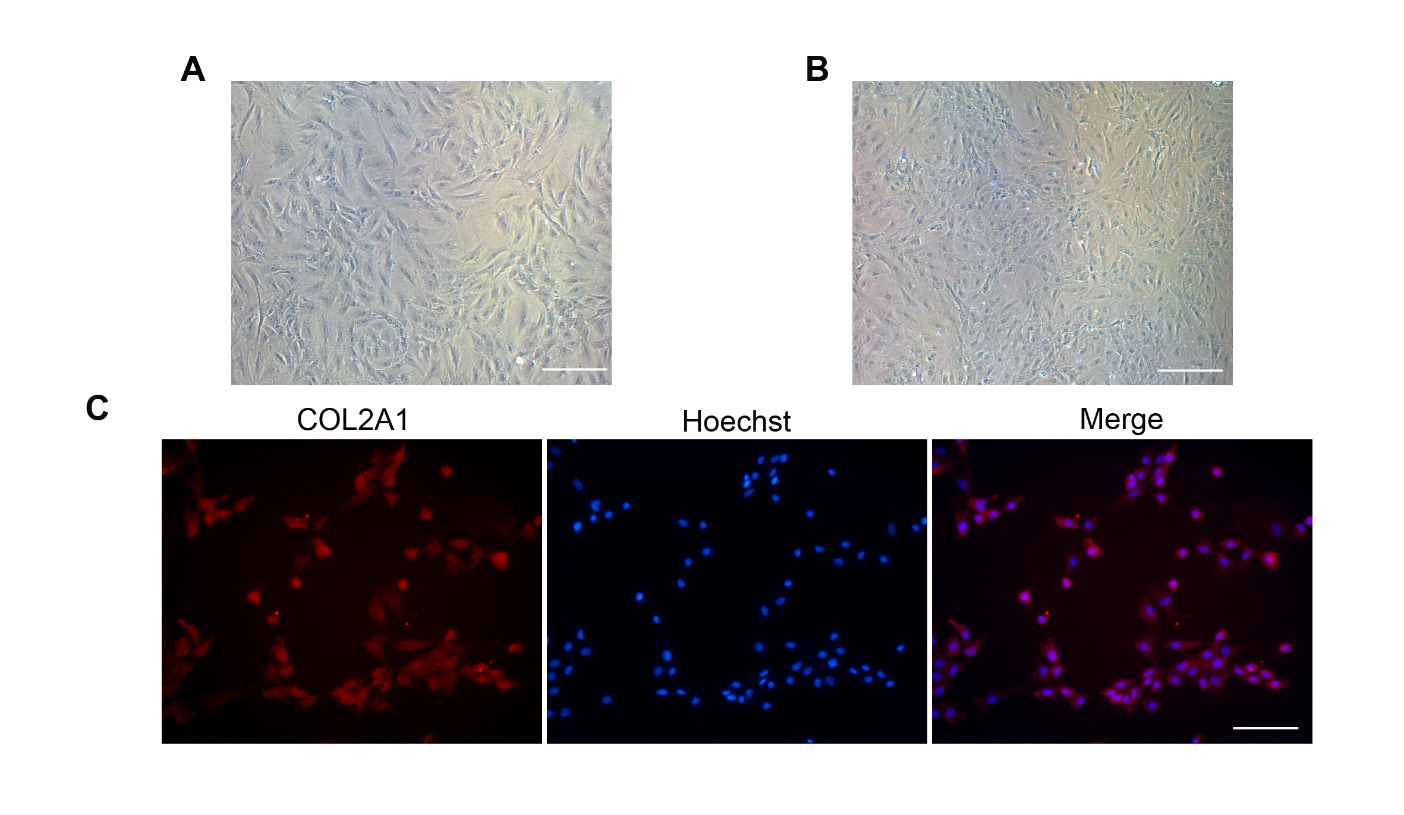


**Figure S2.** Identification of primary rat chondrocytes. (A) Toluidine blue staining of chondrocytes (Scale bars, 200 µm). (B) Alcian blue staining of chondrocytes (Scale bars, 200 µm). (C) Immunofluorescence staining of COL2A1 in chondrocytes (Scale bars, 100 µm).
